# Diurético na Insuficiência Cardíaca Descompensada: Com ou Sem Sal?

**DOI:** 10.36660/abc.20240393

**Published:** 2024-07-31

**Authors:** Wolney de Andrade Martins

**Affiliations:** 1 Universidade Federal Fluminense Niterói RJ Brasil Universidade Federal Fluminense, Niterói, RJ – Brasil

**Keywords:** Solução Salina Hipertônica, Insuficiência Cardíaca, Diuréticos

A síndrome de insuficiência cardíaca (IC) aguda ou descompensada é causa comum de admissões nas salas de emergências ou clínicas de IC. Frequentemente os pacientes se apresentam em perfil “B” da classificação hemodinâmica e têm na baixa adesão terapêutica a causa de sua descompensação. Nesta situação clínica, o emprego de diurético de alça, venoso, em dose alta, tem sido o pilar do tratamento nas últimas cinco décadas. Não muda o prognóstico da doença de base em longo prazo, mas transloca o paciente da crise aguda para a compensação ambulatorial.

Muitas são as questões envolvidas na terapia diurética para a descongestão. Há questionamentos sobre doses; infusão contínua ou *bolus*; parâmetros para a descongestão completa; e os cuidados para evitar hipovolemia. A resistência aos diuréticos e a avidez pelo sódio são mecanismos presentes em pacientes mais graves e crônicos tornando a descongestão ainda mais desafiadora.^1^ Felizmente, algumas soluções aos problemas referidos têm sido propostas e testadas.

A solução salina hipertônica (SSH) ganhou destaque nos anos de 1980 pelos históricos trabalhos dos pesquisadores brasileiros Irineu Tadeu Velasco e Mauricio Rocha e Silva, na ressuscitação do choque hemorrágico.^2^ A SSH popularizou-se nas emergências entre os socorristas das vítimas de acidentes de trânsito. Daí a extrapolar-se ao modelo do paciente com coração disfuncionante foi um desafio! Fundamentos fisiopatológicos fornecem elementos favoráveis e contrários. De um lado o poder osmótico da hipernatremia em atrair água para o compartimento vascular, e por outro a sobrecarga volêmica sobre um coração hipocinético ou com pressão diastólica final elevada. Que lado da balança pesa mais? Benefício ou malefício? O racional fisiopatológico é a base da hipótese clínica, mas somente o ensaio clínico, especialmente se randomizado e controlado, define a recomendação de uma conduta. Então vamos à busca da melhor evidência!

Consensualmente ou não, a adição de SSH à terapia diurética tem sido empregada nos casos de IC avançada. Resta saber se seria uma opção rotineira, de uso inicial, para todo paciente congesto com IC descompensada ou alternativa nos casos não responsivos.

Colluoglu et al.^3^ avaliaram 171 pacientes com IC descompensada, com congestão evidenciada por métodos de imagem, utilizando dados retrospectivos obtidos em prontuários neste artigo publicado nos Arquivos Brasileiros de Cardiologia. Excluíram aqueles com hipoperfusão, hipernatremia ou com episódios de edema agudo de pulmão, síndrome coronariana aguda ou arritmias. Consideraram como desfechos a piora na função renal, a necessidade por diálise, o sucesso na descongestão, a permanência no hospital, as internações por IC e a mortalidade. Procederam à comparação dos 63 pacientes que receberam SSH, duas vezes ao dia até a alta, somados à terapia diurética padrão, com os 108 pacientes que utilizaram exclusivamente diurético.

Em suma, Colluoglu et al.^3^ sinalizam em prol do uso de SSH baseado nos resultados de maior natriurese na segunda hora e no aumento do débito urinário nas primeiras 24 horas, como também nos achados de diminuição do peso corporal e dos níveis de BNP, comuns em ambos os grupos. Houve hemoconcentração no grupo com SSH. Os autores a interpretaram de modo benéfico, pois a relacionam com melhores desfechos. Este achado não é consensual, pois Issa et al. encontraram níveis normais de sódio em trabalho congênere.^4,5^

A natureza retrospectiva dos dados coletados por Colluoglu et al.^3^ e a relativa pequena amostra deste trabalho podem ter comprometido seus resultados. A reprodutibilidade da recomendação rotineira de SSH aos pacientes admitidos com IC descompensada por congestão ainda não ganhou a recomendação consensual das principais diretrizes de IC. Entretanto, revisão recentemente publicada se refere a outros trabalhos clínicos de pequena casuística e, em especial, ao trabalho de mais de 1.900 pacientes inclusos com seguimento médio de 57 meses em que houve redução de desfechos duros como mortalidade e secundários como tempo de hospitalização, redução de peso e aumento da diurese.^6,7^

As dúvidas não terminam por aqui. Outro questionamento é se pacientes ambulatoriais com IC e descompensações frequentes se beneficiariam da adição de SSH ao esquema diurético. Para tentar responder, há em curso o estudo espanhol SALT-HF trial,^7^ cujos resultados são aguardados.

Este trabalho publicado nos Arquivos Brasileiros de Cardiologia,^3^ em conjunto com a literatura, contribui para consolidar a indicação da solução salina hipertônica em adição aos diuréticos para a descongestão dos pacientes com IC descompensada.
