# Supplementary material for: Diurético na Insuficiência Cardíaca Descompensada: Com ou Sem Sal?
Source: Arq Bras Cardiol. 2024 Jul 31;121(7):e20240393. [Article in Portuguese] doi: 10.36660/abc.20240393 (PMC11341204; doi:10.36660/abc.20240393)
Supplement: Translation

# Translation: Diuretic in Decompensated Heart Failure: With or Without Salt?

**DOI:** 10.36660/abc.20240393i

**Authors:** Wolney de Andrade Martins

**Affiliations:** 1 Brazil Universidade Federal Fluminense, Niterói, RJ – Brazil

**Keywords:** Hypertonic Saline Solution, Heart Failure, Diuretics

Acute or decompensated heart failure (HF) syndrome is a common cause of admission to emergency rooms or HF clinics. Patients often present in profile “B” of the hemodynamic classification and have low therapeutic adherence as the cause of their decompensation. In this clinical situation, the use of high-dose intravenous loop diuretics has been the mainstay of treatment over the last five decades. It does not change the long-term prognosis of the underlying disease, but it moves the patient from the acute crisis to outpatient compensation.

There are many issues involved in diuretic therapy for decongestion. There are questions about doses; continuous infusion or *bolus*; parameters for complete decongestion; and care to avoid hypovolemia. Resistance to diuretics and sodium craving are mechanisms present in more severe and chronic patients, making decongestion even more challenging.^1^ Fortunately, some solutions to the problems mentioned above have been proposed and tested.

Hypertonic saline solution (HSS) gained prominence in the 1980s due to the historic work of Brazilian researchers Irineu Tadeu Velasco and Mauricio Rocha e Silva, in the resuscitation of hemorrhagic shock.^2^ HSS became popular in emergencies among rescuers of victims of traffic accidents. Therefore, extrapolating to the model of the patient with a dysfunctional heart was a challenge! Pathophysiological backgrounds provide favorable and contrary elements. On the one hand, the osmotic power of hypernatremia in attracting water to the vascular compartment, and on the other, the volume overload on a hypokinetic heart or with high end-diastolic pressure. Which side of the scale weighs more? Benefit or harm? The pathophysiological rationale is the basis of the clinical hypothesis, but only the clinical trial, especially if randomized and controlled, defines the recommendation for a course of action. So let’s look for the best evidence!

Consensually or not, the addition of HSS to diuretic therapy has been used in cases of advanced HF. It remains to be seen whether it would be a routine option, for initial use, for all congested patients with decompensated HF or an alternative in non-responsive cases.

Colluoglu et al.^3^ evaluated 171 patients with decompensated HF, with congestion evidenced by imaging methods, using retrospective data obtained from medical records in this article published in *Arquivos Brasileiros de Cardiologia*. They excluded those with hypoperfusion, hypernatremia or episodes of acute lung edema, acute coronary syndrome, or arrhythmias. They considered as outcomes worsening of renal function, the need for dialysis, success in decongestion, hospital stay, hospitalizations for HF, and mortality. They compared 63 patients who received HSS twice a day until discharge, in addition to standard diuretic therapy, with 108 patients who used diuretics exclusively.

In summary, Colluoglu et al.^3^ signal in favor of the use of HSS based on the results of greater natriuresis in the second hour and increased urinary output in the first 24 hours, as well as findings of decreased body weight and BNP levels, common in both groups. There was hemoconcentration in the HSS group. The authors interpreted it beneficially, as they relate it to better outcomes. This finding is not consensual as Issa et al. found normal sodium levels in similar work.^4,5^

The retrospective nature of the data collected by Colluoglu et al.^3^ and the relatively small sample of this work may have compromised its results. The reproducibility of routinely recommending HSS to patients admitted with decompensated HF due to congestion has not yet gained consensus recommendation from the main HF guidelines. However, a recently published review refers to other clinical studies involving small samples and, in particular, the work of more than 1,900 patients included with an average follow-up of 57 months, in which there was a reduction in hard outcomes such as mortality and secondary outcomes such as length of hospitalization, weight reduction and increased diuresis.^6,7^

Another question is whether outpatients with HF and frequent decompensation would benefit from the addition of HSS to the diuretic regimen. To try to answer, the Spanish SALT-HF trial is currently underway,^7^ the results of which are awaited.

This work now published in *Arquivos Brasileiros de Cardiologia*,^3^ together with the literature, contributes to consolidating the indication of hypertonic saline solution in addition to diuretics for the decongestion of patients with decompensated HF.
